# Alexithymia Has No Clinically Relevant Association With Outcome of Multimodal Treatment Tailored to Needs of Patients Suffering From Somatic Symptom and Related Disorders. A Clinical Prospective Study

**DOI:** 10.3389/fpsyt.2018.00292

**Published:** 2018-07-24

**Authors:** Lars de Vroege, Wilco H. M. Emons, Klaas Sijtsma, Christina M. van der Feltz-Cornelis

**Affiliations:** ^1^Department Tranzo, Tilburg School of Social and Behavioral Sciences, Tilburg University, Tilburg, Netherlands; ^2^Clinical Centre of Excellence for Body, Mind and Health, GGz Breburg, Tilburg, Netherlands; ^3^Department of Methodology and Statistics, Tilburg School of Social and Behavioral Sciences, Tilburg University, Tilburg, Netherlands; ^4^Department of Health Sciences, Hull York Medical School, University of York, York, United Kingdom

**Keywords:** alexithymia, treatment outcome, somatic symptom and related disorders, depression, anxiety, physical symptoms, general functioning

## Abstract

**Introduction:** Alexithymia may moderate the effectiveness of treatment and may predict impaired general functioning of patients suffering from somatic symptom and related disorders (SSRD).

**Aim:** We compared alexithymia levels in a clinical prospective study with 234 consecutive patients suffering from SSRD from the Centre of Excellence for Body, Mind, and Health, Tilburg using the Bermond-Vorst Alexithymia Questionnaire, with general population norm scores. Second, we explored treatment outcomes of a multimodal treatment tailored to patient needs by Shared Decision Making (SDM) and Patient Related Outcome Monitoring (PROM) in patients with SSRD. Third, we explored whether alexithymia is associated with treatment outcome. Fourth, we explored if the presence of a chronic medical condition (e.g., diabetes mellitus, cardiovascular diseases) affects the association of alexithymia with treatment outcomes.

**Results:** Compared to norm scores, SSRD patients showed elevated scores on the subscales identifying, verbalizing, and fantasizing, and on the cognitive dimension. All patients benefited from treatment in terms of anxiety, depression, and physical symptoms. The association of alexithymia with treatment outcome was significant, but the effect size was negligible (range odds ratios 1.02–1.25). The association between alexithymia and treatment outcome was stronger in patients suffering from chronic medical conditions compared to patients without chronic medical conditions. However, the effect size of this association was negligible (range odds ratio 0.94–1.12).

**Discussion:** Alexithymia scores are elevated in patients with SSRD compared to general population scores, but the level of alexithymia has no clinically relevant association with treatment outcome both in SSRD patients with and without comorbid chronic medical conditions.

## Introduction

Nemiah and Sifneos (1) introduced the concept of alexithymia to describe an emotional deficiency in patients with classic psychosomatic disorders, such as asthma, and hypertension. Patients were unaware of their feelings or were incapable to verbalize them, and they were unable to fantasize about their inner thoughts, feelings, and attitudes. Although the concept originated from psychoanalytical research, in time it also incorporated other perspectives, such as those originating from cognitive behavioral research and from stress research. In the 1990s, alexithymia was described as a combination of the following features: (a) difficulty identifying and describing feelings, (b) difficulty distinguishing feelings and bodily sensations caused by emotional arousal, (c) constricted imaginal processes, and (d) a cognitive style characterized by a preoccupation with the details of external events (2). These characteristics are related to stress and adaptation, and have repercussions for psychotherapeutic treatment possibilities.

Although alexithemic patients were prepared to participate in therapy consisting of a psychodynamic oriented multimodal therapy (3), alexithymia has also been described as interfering with psychotherapy (4), such as group psychotherapy, individual psychodynamic psychotherapy, and supportive therapy. Recent studies found that specifically focusing on alexithymia during treatment improved treatment outcomes in terms of symptom reduction and general functioning (5–9), but the results of a 2013 systematic review (10) were inconclusive, and was suggested that development of evidence-based treatments are necessary (10). Since most psychotherapeutic approaches rely on the patients' access to their emotions, patients unable to address these emotions provide a challenge for therapists. We do not know of studies exploring the association between alexithymia and treatment outcome at symptom level in patients suffering from somatic symptom and related disorder (SSRD).

Because alexithymia was found to be related to impoverished general functioning in somatoform disorders (2, 11–21), following the classification of the Diagnostic and Statistical Manual of Mental Disorders (DSM)-IV-TR (22) and other precursors of SSRD, as described in DSM-5 (23), general functioning also may be a relevant outcome of treatment. As far as we know, studies exploring this aspect of treatment outcome in patients suffering from SSRD have not been done. This study explores whether alexithymia has a moderating effect in treating depressive, anxiety, physical symptoms, and general functioning in SSRD patients.

## Objectives and hypotheses

The first objective of this study was to estimate the level of alexithymia of patients suffering from SSRD and compare this level to known norm scores for the general population. We expected the alexithymia scores of SSRD patients to be higher.

The second objective was to explore outcomes of a multimodal treatment tailored to patient needs by Shared Decision Making (SDM) and Patient Related Outcome Monitoring (PROM) (24) with respect to depression, anxiety, physical symptoms, and general functioning in patients with SSRD, independent of alexithymia scores at baseline. We expected the treatment outcome to be improved.

The third objective was to examine the association of alexithymia with treatment outcomes with respect to depression, anxiety, physical symptoms, and general functioning. We predicted the patients with high levels of alexithymia show less favorable treatment outcomes than patients with low levels of alexithymia.

The fourth objective was to examine the influence of chronic medical conditions (e.g., diabetes mellitus, cardiovascular diseases) on the association between alexithymia and treatment outcome. We hypothesized a stronger association between alexithymia and treatment outcomes in patients suffering from chronic medical condition and alexithymia than in patients without chronic medical condition.

## Methods

### Study design

The study uses data from a longitudinal observational design in a clinical setting. The sample existed of patients suffering from SSRD who were treated at the Clinical Centre of Excellence for Body, Mind and Health (Dutch abbreviation: CLGG), a department of GGz Breburg, Tilburg, the Netherlands. We assessed alexithymia at intake, and we assessed outcome measures including depression, anxiety, physical symptoms, and general functioning at intake before treatment and at discharge. All patients who were referred to CLGG between August 2013 and April 2016 were included in the study.

The standard intake procedure at the CLGG consists of questionnaire assessment during intake (referred to as baseline measurement), case history assessment, physical assessment, psychiatric evaluation, and psycho-diagnostic assessment. The Bermond-Vorst Alexithymia Questionnaire (BVAQ) was self-administered during the psycho-diagnostic assessment at intake. Level of education was determined using Verhage coding (25), which includes seven levels ranging from low (levels 1 through 4), medium (level 5) to high (levels 6–7). Throughout treatment, patient's progress was evaluated using computerized Routine Outcome Monitoring (ROM) (26). We used ROM data with regard to depression, anxiety, physical symptoms and general functioning scores. Patients were informed at intake about the scientific research conducted at CLGG. Patients who did not give their consent to use their data were excluded from the dataset. Data were coded in order to create an anonymous dataset. The Commission of Scientific Research of GGz Breburg approved of this study (file number: CWO 2014-09).

### Setting and participants

Inclusion/exclusion criteria were evaluated for all patients that were referred to CLGG. Inclusion criteria were (1) completion of the intake, and (2) age at least equal to 18 years. Patients were excluded if they (1) were engaged in personal or professional injury procedures, (2) were not able to come to CLGG, (3) did not complete questionnaires from the ROM during intake and during treatment, and (4) had an IQ below 80 [assessed during intake using the Dutch Adult Reading Test (27)]. In addition, they were excluded (5) if the primary treatment focus was not related to physical symptoms. Other exclusion criteria were (6) presence of psychosis or psychotic features that hampered treatment, (7) an active suicide risk (threatening), and (8) substance dependency. Comorbid conditions and DSM-5 classifications were assessed in a clinical interview during intake.

Treatment at CLGG was of multimodal, in accordance with the multidisciplinary guideline for medically unexplained symptoms and somatic disorders (28, 29), and tailored to the needs and treatment expectations of the patient. Treatment consisted of cognitive behavioral therapy (CBT), acceptance and commitment therapy (ACT), or problem solving treatment (PST) provided by trained psychologists, in combination with pharmacotherapy provided by a physician or psychiatrist. The psychotherapeutic treatments were provided sequentially and were tailored to the needs and treatment expectations of the patients. During treatment, every 3 months both psychotherapeutic and pharmacotherapeutic treatment were adjusted based on progress in terms of PROM (24) and in a SDM (25) by multidisciplinary team consultation. Patients were treated for 1 year on average, using this multimodal approach.

**Figure 1:**
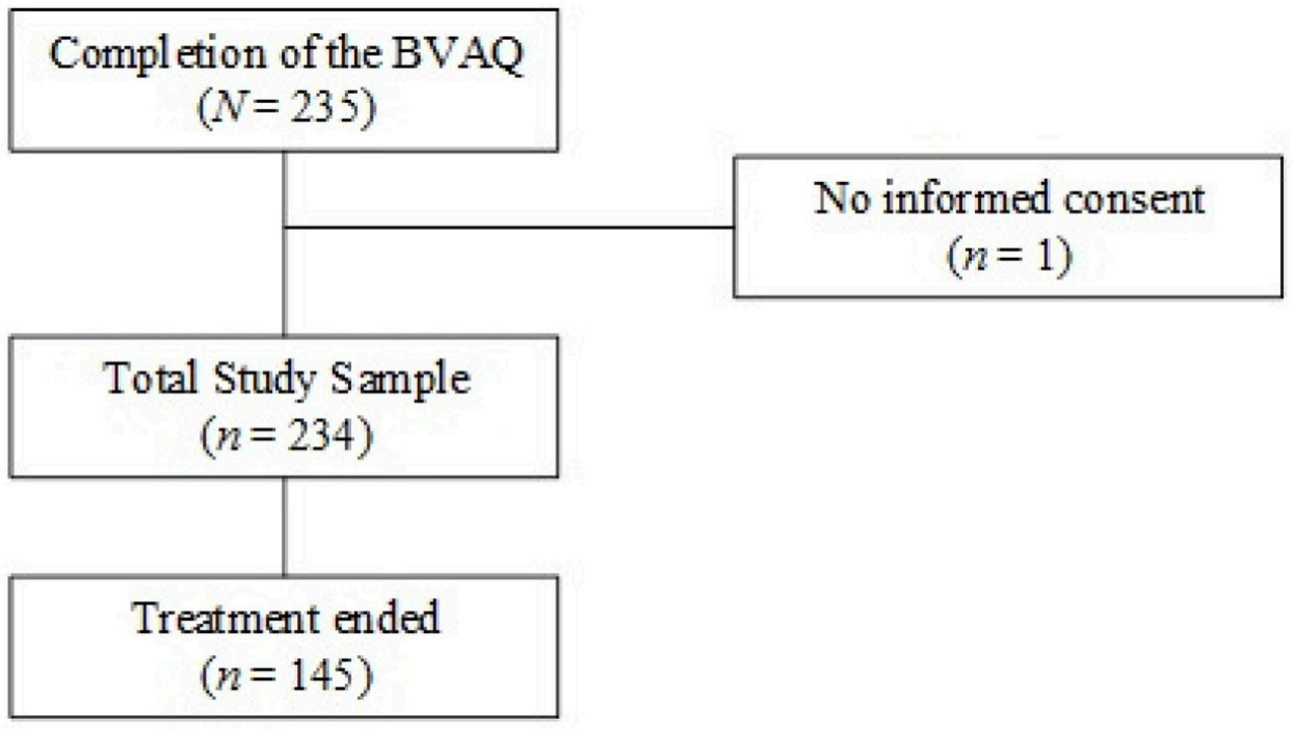
Flowchart of patients included in the study. Sample sizes are given for patients who completed treatment and questionnaire assessment. BVAQ, Bermond-Vorst Alexithymia Questionnaire.

Figure 1 shows a flow chart of the study. Two hundred and thirty-five patients filled out the BVAQ at intake. One patient, who gave no consent, was excluded from the study. Of the remaining 234 patients, 145 (62.0%) completed treatment. Of the patients who completed treatment, 142 patients (97.9%) filled out the Physical Symptom Checklist (PSC), 142 (97.9%) filled out the Generalized Anxiety Disorder questionnaire (GAD-7), 144 (99.3%) filled out the Patient Health Questionnaire for assessing depression (PHQ-9), and 126 (86.9%) filled out the 36-item Short Form Health Survey (SF-36), both at intake and at discharge.

### Instruments

We assessed depression, anxiety, physical symptoms, and general functioning before, during and after treatment by means of questionnaires with PROM. Alexithymia was only assessed at intake.

#### Bermond-vorst alexithymia questionnaire (BVAQ)

Alexithymia was assessed using the 40-item BVAQ (30). The BVAQ provides valid and reliable measures of cognitive and affective dimensions of alexithymia (30–34). The Toronto Alexithymia Scale (35, 36), another instrument widely used in alexithymia research, covers cognitive factors of alexithymia but not affective dimensions. We preferred the BVAQ because it has a broader scope.

Responses to the items were scored on a five-point Likert scale. Higher scores indicate higher levels of alexithymia. The BVAQ consists of five subscales containing eight items each. The subscales are identifying, verbalizing, analyzing, fantasizing, and emotionalizing, each in accordance with the five-factor model of alexithymia (30). The five subscales constitute a cognitive dimension and an affective dimension. Scores on the cognitive dimension were obtained by adding the scores of the subscales identifying, analyzing, and verbalizing (scores ranged from 24 through 120). Scores on the affective dimension were obtained by adding the scores of the subscales emotionalizing and fantasizing (scores ranged from 16 through 80). In our SSRD study sample, coefficient alpha (37) for the cognitive dimension equaled 0.90 and for the affective dimension it equaled 0.68.

#### The patient health questionnaire (PHQ-9)

Depression was assessed using the PHQ-9 (38). The PHQ-9 is a reliable 9-item self-report questionnaire, with higher scores indicating higher levels of depressive symptoms (38). Item scores ranged from 0 (not at all) to 3 (nearly every day), and total scores ranged from 0 to 27 (38). Cutoff points of 5, 10, 15, and 20 represent mild, moderate, moderately severe and sever levels of depression (39).

#### Generalized anxiety disorder questionnaire (GAD-7)

Anxiety was assessed using the GAD-7. The GAD-7 is a reliable 7-item self-report questionnaire that measures symptoms of anxiety during the last 2 weeks (40). GAD-7 scores range from 0 to 21, and cutoff scores of 5, 10, and 15 represent mild, moderate and severe levels of anxiety (39).

#### Physical symptom checklist (PSC)

Physical symptoms were measured using the PSC (41), which is a 51-item questionnaire. The total score on the PSC ranges from 0 to 51 and represents the number of physical symptoms that were regularly or often present in the last week (41). De Waal and Van Hemert (42) provided normative data.

#### 36-item short form health survey (SF-36)

We used the SF-36 (43) to assess general functioning. Studies confirmed the SF-36's validity and reliability (44–46). The SF-36 is a self-report questionnaire that contains 36 items, which are distributed across eight scales. Using the developers' scoring algorithm (47), the eight subscales were converted into two summary measures, a physical component summary measure (PCS) and a mental component summary measure (MCS). Scores range from 0 to 100, where higher scores on the PCS and MCS indicate better general functioning. Normative data are available in Maglinte, Hays (48).

### Treatment outcome variables

#### Raw change

For each outcome, a change score variable was created by subtracting the score after treatment from the scores at intake. This way, change scores represented treatment outcomes with respect to depression, anxiety, physical symptoms, and general functioning (PCS and MCS).

#### Reliable change

To examine in more detail to what extent alexithymia predicts reliable changes at the individual level, we adopted Jacobson and Truax' (49) framework. Using their reliable change index (RCI), we determined which patients showed reliable change on the PHQ-9, the GAD-7, the PSC, the PCS of the SF-36, or the MCS of the SF-36. A dichotomous change variable was created, reflecting change at a 90% confidence level; that is, scores equal to 0 reflected no reliable change (i.e., −1.645 < RCI < 1.645) and scores equal to 1 reflected reliable change (i.e., RCI < −1.645 or RCI > 1.645).

#### Clinical change

To further study the association between alexithymia and clinical change at the individual level, we defined a categorical variable called clinical remission. A patient shows clinical remission if change is reliable and his/her score at intake exceeds a clinical cutoff but not any more after the treatment. The following clinical cutoffs were used to define remission. For both the PHQ-9 and GAD-7, we used a score of 5, which identifies at least mild levels of depression or anxiety. For the PSC, we also used 5 as the cutoff. This cutoff coincides with the 75th percentile of PSC scores in normative data from general practitioner's offices (42). This means that remission is observed if after the treatment the patient's PSC score is no longer among the highest 25% in the general population. To define remission on the PCS and MCS of the SF-36, the mean scores in the general population were used (48). In particular, the cutoffs for remission were 50 for the PCS and 54 for the MCS after treatment. Furthermore, to speak of clinical remission, patients must also have shown reliable change. This results in a clinical change variable having three levels: 0 = no reliable change (i.e., |RCI| < 1.645), 1 = reliable change but no remission, and 2 = remission.

### Statistical methods

*Objective 1:* Level of alexithymia was described by means of normed scores. These normed scores were obtained using normative data from the general population (50). In particular, normed scores have a mean zero and a standard deviation equal to 1. Positive norm scores suggest above average levels compared to the general population. We used one-sample *t*-tests to test whether mean differences between patients suffering from SSRD and the normal group with respect to the normed scores were significant.

*Objective 2:* We studied mean differences between the raw scores at intake and at discharge for the PHQ-9, GAD-7, PSC, and PCS and MCS of the SF-36, using the paired-sample *t*-tests, for the complete group. For each outcome measure, effect-size Cohen's *d* was obtained following Rosner (51). Effect sizes equal to *d* = 0.2 are considered small, *d* = 0.5 medium, and *d* ≥ 0.8 large (52).

*Objective 3:* We used linear regression analysis to explore the association of alexithymia with depression, anxiety, physical symptoms, and general functioning. The raw change-score variables for the PHQ-9, the GAD-7, the PSC, the PCS of the SF-36, and the MCS of the SF-36 were used as dependent variables.

We used multinomial logistic regression to explore the association of alexithymia with clinical change for depression, anxiety, physical symptoms, and general functioning. Logistic regression and multinomial regression analysis provide insight into the predictive value of alexithymia in the clinical context.

*Objective 4:* Chronic medical condition and the two interaction terms of the dimension of alexithymia with chronic medical condition were used as independent variables in regression models.

We used logistic regression to investigate if the cognitive and affective dimensions, chronic medical condition, and the interactions between the dimensions of alexithymia and chronic medical condition predict reliable change at the individual level. For each outcome variable, the regression analyses were done as follows. First, we estimated the full model that included as predictors the background variables (age, gender, and education level), the first-order effects of the cognitive and affective alexithymia dimensions, and chronic medical conditions, and the interaction effects between the alexithymia dimensions and medical conditions. To study the interaction effects, we centered the independent variables to avoid potential problems with respect to multicollinearity (53). Second, in case some of the interaction effects were non-significant, we re-estimated the model without the non-significant interaction effects.

For logistic regression analysis, we used Nagelkerke's *R*-square to gauge effect size. Formally, the pseudo *R*-square does not represent proportions of explained variance, but we interpreted the pseudo *R*-square as if it did provide the proportion of the variation the model explained (54). We used the guidelines of Cohen (**?**) to interpret Nagelkerke's pseudo *R*-square (i.e., *R*-square = 0.02 was considered small, *R*-square = 0.13 was considered medium, *R*-square ≥ 0.26 was considered large). All analyses were done by means of the Statistical Package for the Social Sciences version 22 (55).

## Results

### Sample characteristics

Two hundred and two patients (86.3%) were diagnosed with a somatic symptom disorder, 10 patients (4.3%) were diagnosed with an illness anxiety disorder, and 22 patients (9.4%) were diagnosed with a conversion disorder.

Table 1 (upper panel) describes the socio-demographic characteristics of the SSRD patient sample. The SSRD sample consisted of 234 patients (59.0% females). The sample had a mean (*M*) age of 42.8 [standard deviation (*SD*) = 12.56; range: 19 to 79]. Seven patients had missing values on the BVAQ items. One of these patients had six missing item scores and was excluded from further analyses. The only missing item score for the remaining six patients was imputed using two-way imputation (56, 57).

**Table 1:** Socio-demographic characteristics and descriptive statistics for the BVAQ in the SSRD sample at intake (*N* = 234).

**Characteristic**	***M* (*SD*)**	**Min/Max**	***n* (*%*)**	**Normed scores*^a^* (min/max)**	***p***
**BACKGROUND VARIABLES**					
Gender					
Men			96 (41.0)		
Women			138 (59.0)		
Age	42.78 (12.56)	19/79			
Educational level^*^					
Low (1–4)			56 (24.9)		
Medium (5)			103 (45.8)		
High (6–7)			66 (29.2)		
(Missing value)			(9)		
Marital status					
Married/living together			150 (71.4)		
Divorced			11 (5.2)		
Widow(er)			1 (0.5)		
Single			48 (22.9)		
(Missing value)			(24)		
PSC	16.57 (8.08)	0/38			
GAD-7	11.51 (5.47)	0/21			
PHQ-9	14.21 (6.07)	0/27			
SF-36 (*n* = 225)^*b*^					
PCS	40.48 (5.44)	27.49/57.43			
MCS	44.01 (5.16)	21.30/55.55			
Comorbidity at intake					
Comorbid anxiety			2 (0.9)		
Comorbid depression			25 (11.4)		
Comorbid depression and anxiety			193 (87.7)		
**BVAQ SCORES**					
Cognitive dim.	67.12 (17.64)	32/106		0.43 (−2.38/3.25)	< 0.001
Identifying	22.10 (7.02)	8/40		0.69 (−1.97/4.26)	< 0.001
Analyzing	19.61 (6.02)	8/34		−0.04 (−2.52/2.97)	0.618
Verbalizing	25.41 (8.29)	8/40		0.39 (−2.87/3.08)	< 0.001
Affective dim.	45.48 (8.88)	19/66		−0.10 (−2.79/2.45)	0.104
Fantasizing	26.74 (6.92)	8/40		0.27 (−2.51/2.42)	< 0.001
Emotionalizing	18.74 (5.16)	8/32		−0.55 (−2.96/2.48)	< 0.001

SSRD, Somatic Symptom and Related Disorders; PSC, Physical Symptom Checklist; GAD-7, Generalized Anxiety Disorder questionnaire; PHQ-9, Patient Health Questionnaire; SF-36, 36-item Short Form Health Survey; MCS, Mental Component Summary; PCS, Physical Component Summary; BVAQ, Bermond-Vorst Alexithymia Questionnaire.

a Normed scores were based on normative data (50);

b *225 participants of the whole sample completed the SF-36*.

* Using Verhage coding (25).

*The level of alexithymia of patients suffering from SSRD compared to known norm scores for the general population*.

Table 1 (lower panel) shows the means for the raw scores (column 2) and normed scores (column 5) on the BVAQ. Using a significance level equal to 0.007 (alpha of 0.05 divided by 7, equal to the number of used tests), significant mean differences with respect to the norm scores were found for the subscales verbalizing [*t*(233) = 4.239, *p* < 0.001], fantasizing [*t*(233) = 3.770, *p* < 0.001], identifying [*t*(233) = 7.759, *p* < 0.001], and emotionalizing [*t*(233) = −8.106, *p* < 0.001]. A significant mean difference was found for the cognitive dimension [*t*(233) = 4.944, *p* < 0.001]. For the subscales of the cognitive dimension, we found elevated levels of identifying (*M* = 0.69; range −1.97 to 4.26) and verbalizing (*M* = 0.39; range −2.87 to 3.08) compared to the general population. No significant mean differences were found between SSRD patients and the general population for analyzing [*t*(233) = −0.500, *p* = 0.618] and the affective dimension [*t*(233) = −1.632, *p* = 0.104]. For the subscales of the affective dimension in the BVAQ, we found lowered levels of emotionalizing (*M* = −0.55; range −2.96 to 2.48), but higher mean values for fantasizing (*M* = 0.27; range −2.51 to 2.42).

*Treatment outcomes of a multimodal treatment tailored to patients needs by SDM and PROM in terms of depression, anxiety, physical symptoms, and general functioning*.

Table 2 shows the mean scores before and after treatment for the PSC, GAD-7, PHQ-9, and the MCS and PCS of the SF-36. Results suggested substantive mean improvements of the treatment outcomes. PSC means before and after treatment improved significantly [*t*(141) = 4.207, *p* < 0.001, *d* = 1.82], the mean scores on the PHQ-9 also differed significantly before and after treatment [*t*(143) = 4.837, *p* < 0.001, *d* = 1.43], and the mean scores on the GAD-7 differed significantly before and after treatment [*t*(141) = 5.090, *p* < 0.001, *d* = 1.21]. Mean MCS and PCS scores for the SF-36 did not differ significantly before and after treatment [respectively, *t*(125) = 0.757, *p* = 0.450, *d* = 0.92 and *t*(126) = 1.494, *p* = 0.138, *d* = 1.06].

**Table 2:** Mean scores on the PHQ-9, GAD-7, PSC, and SF-36 of the SSRD sample at intake and after treatment.

**Outcome measure**	***N***	**Measurement occasion**				
		**At intake**		**After treatment**		
		***M***	***SD***	***M***	***SD***	***p***
PSC	142	16.26	7.68	13.54	9.22	< 0.001
GAD-7	142	11.18	5.41	9.09	6.17	< 0.001
PHQ-9	144	13.94	6.12	11.50	7.36	< 0.001
SF-36	126					
PCS		41.10	5.44	40.42	5.48	0.138
MCS		43.47	5.73	43.03	5.82	0.450

M, Mean; SD, Standard Deviation; PHQ-9, Patient Health Questionaire-9; GAD-7, General Anxiety Disorder questionnaire; PSC, Physical Symptom Checklist; SF-36, 36-item Short Form Health Survey; MCS, Mental Component Summary; PCS, Physical Component Summary.

*n = the number of patients who completed the treatment and who filled out the questionnaire both at intake and after treatment*.

*The association of alexithymia with treatment outcomes in terms of depression, anxiety, physical symptoms, and general functioning*.

Alexithymia had significant association with treatment outcome regarding anxiety (Table 3). The cognitive and affective dimensions did not significantly predict change with respect to depression, physical symptoms scores, and general health functioning.

**Table 3:** Linear regression of raw change scores for the GAD-7 on the BVAQ dimensions and covariates.

**Predictors**	**Change scores for the GAD-7**		
	***B***	**95% CI**	***P***
Cognitive dimension	−**0.08**	**[**−**0.15**, −**0.01]**	**0.021**
Affective dimension	−0.01	[−0.13, 0.12]	0.911
Chron med cond	0.76	[−0.97, 2.49]	0.386
Int cogn_med	**0.12**	**[0.02, 0.22]**	**0.022**
Int aff_med	−0.15	[−0.35, 0.05]	0.140

GAD-7, Generalized Anxiety Disorder questionnaire; 95% CI, 95% confidence interval for B; Chron med cond, chronic medical condition; Int cogn_med, interaction between the cognitive dimension of alexithymia and chronic medical condition; Int aff_med, interaction between the affective dimension and chronic medical condition.

*All coefficients in bold are significant at the 5% significance level*.

Table 4 shows the results of the logistic regression analyses. The cognitive dimension had a significant negative main association on treatment outcome with respect to anxiety (*Odds Ratio* (*OR*) = 1.02, 95*%CI* = [1.00, 1.05]). The squared semi-partial correlation for this dimension was 0.05, which means that 5% of the total variability of the treatment outcome for anxiety is uniquely associated with the cognitive dimension. The affective dimension was associated with a significant positive association on treatment outcome with respect to general mental health functioning (*OR* = 1.25, 95*%CI* = [1.09, 1.44]). Removing the affective dimension decreased *R*-square to .15, which renders the association substantial.

**Table 4:** Logistic regression analyses predicting reliable change regarding depression, physical symptoms, and general functioning.

**Predictors**	**Outcome variable**											
	**Depression (PHQ-9)**			**Anxiety (GAD-7)**			**Physical symptoms (PSC)**			**General functioning (MSC of the SF-36)**		
	***OR***	**95% CI**	***R*^2*a*^**	***OR***	**95% CI**	***R^2*a*^***	***OR***	**95% CI**	***R^2*a*^***	***OR***	**95% CI**	***R^2*a*^***
			0.10			0.08			0.14			0.43
Cogn dim	1.03	[1.00, 1.06]		**1.02**	**[1.00, 1.05]**		1.01	[0.98, 1.04]		1.00	[0.96, 1.04]	
Aff dim	1.03	[0.98, 1.09]		0.98	[0.94, 1.03]		0.99	[0.94, 1.05]		**1.25**	**[1.09, 1.44]**	
Chron med cond	1.06	[0.50, 2.26]		0.93	[0.45, 1.92]		1.38	[0.63, 2.98]		0.42	[0.08, 2.28]	
Cogn × medical condition	**0.95**	**[0.91, 0.99]**		–	–		**0.95**	**[0.91, 1.00]**		–	–	
Aff × medical condition	1.01	[0.92, 1.10]		–	–		1.10	[1.00, 1.21]		–	–	

PHQ-9, Patient Health Questionnaire; GAD-7, Generalized Anxiety Disorder questionnaire; PSC, Physical Symptom Checklist; MCS, Mental Component Summary; SF-36, 36-item Short Form Health Survey; OR, Odds Ratio; 95% CI, 95% confidence interval; Chron med cond, chronic medical condition; Int cognmed, interaction term of the cognitive dimension of alexithymia and chronic medical condition; Int aff, interaction term of the affective dimension and chronic medical condition.

Results for chronic medical condition, and for the interaction terms of the alexithymia dimension and chronic medical condition (Model 3 and 4; respectively) yielded no significant results.

a *Nagelkerke's pseudo R-square*.

*All coefficients in bold are significant at the 5% significance level*.

Table 5 shows the results for predicting reliable and clinical change. With regard to general functioning (MCS of the SF-36), the affective dimension was significantly associated with clinical change in the group of patients with no remission vs. the group of patients with no clinical change and no remission (*OR* = 1.24, 95*%CI* = [1.08, 1.42]). Results for clinical change and remission vs. no clinical change and no remission could not be computed, because none of the patients showed remission on the MCS. No significant associations were found between anxiety and for the PCS of the SF-36.

**Table 5:** Multinomial logistic regression analyses predicting clinical change regarding depression, physical symptoms, and general functioning.

**Predictors**	**Depression (PHQ-9)**			**Physical symptoms (PSC)**			**General functioning (MCS of the SF-36)**		
	***OR***	**95% CI**	***p***	***OR***	**95% CI**	***p***	***OR***	**95% CI**	***p***
**CLINICAL CHANGE/NO REMISSION vs. NO CLINICAL CHANGE/NO REMISSION**									
Affective dimension	1.02	[0.95, 1.09]	0.561	1.00	[0.94, 1.06]	0.880	**1.24**	**[1.08, 1.42]**	**0.003**
Cognitive dimension	1.03	[1.00, 1.07]	0.091	1.01	[0.98, 1.04]	0.572	0.90	[0.95, 1.04]	0.630
Chron med cond	0.86	[0.33, 2.22]	0.751	1.25	[0.53, 2.94]	0.618	0.39	[0.07, 2.12]	0.276
Int aff_med	1.00	[0.89, 1.11]	0.944	**1.12**	**[1.00, 1.24]**	**0.048**	–	–	–
Int cogn_med	**0.94**	**[0.89, 0.99]**	**0.021**	0.95	[0.91, 1.00]	0.054	–	–	–
**CLINICAL CHANGE/REMISSION vs. NO CLINICAL CHANGE/NO REMISSION**									
Affective dimension	1.03	[0.95, 1.12]	0.424	1.01	[0.90, 1.15]	0.822	–	–	–
Cognitive dimension	1.02	[0.98, 1.07]	0.301	1.00	[0.94, 1.07]	0.977	–	–	–
Chron med cond	1.46	[0.52, 4.12]	0.613	1.19	[0.22, 6.40]	0.836	–	–	–
Int aff_med	0.97	[0.91, 1.02]	0.230	1.03	[0.86, 1.25]	0.732	–	–	–
Int cogn_med	1.03	[0.91, 1.16]	0.643	0.95	[0.87, 1.05]	0.319	–	–	–

PHQ-9, Patient Health Questionnaire; PSC, Physical Symptom Checklist; MCS, Mental Component Summary; SF-36, 36-item Short Form Health Survey; OR, Odds Ratio; 95% CI, 95% confidence interval; Chron med cond, chronic medical condition; Int cogn_med, interaction term of the cognitive dimension of alexithymia and chronic medical condition; Int aff_med, interaction term of the affective dimension and chronic medical condition.

*Results not reported because interaction effect was not significant, or effect could not be estimated because no patients showed remission on the MCS*.

*All coefficients in bold are significant at the 5% significance level*.

*Influence of chronic medical conditions (e.g., diabetes mellitus, cardiovascular diseases, and others) on the association between alexithymia and treatment outcome*.

The cognitive dimension and medical condition showed a significant interaction association (see Table 3). Simple effects analysis suggested a negative association for patients without a chronic medical condition (*B* = −0.08, *p* = 0.022), and a non-significant association for patients with a chronic medical condition (*B* = 0.04, *p* = 0.329).

The interaction between the cognitive dimension and chronic medical condition had a significant association with reliable change with regard to depression (*OR* = 0.95, 95*%CI* = [0.91, 0.99]) (Table 4). The squared semi-partial correlation for this interaction was 0.06, which means that 6% of the total variability of treatment outcome for depression is uniquely associated with the interaction between cognitive dimension of alexithymia and chronic medical condition. The OR equal to 0.95 suggests that the association between alexithymia (cognitive dimension) and treatment outcome in terms of depression is negative for patients with chronic medical condition compared to patients without a chronic medical condition. Nevertheless, these correlations and ORs are very small and we render them clinically irrelevant.

Regarding physical symptoms, a significant interaction effect between cognitive dimension and chronic medical condition was found for the PSC (*OR* = 0.95, 95*%CI* = [0.91, 1.00]). The OR equal to 0.95 suggests that the association between alexithymia (cognitive dimension) and treatment outcome in terms of physical symptoms is negative for patients with chronic medical condition compared to patients without a chronic medical condition. The squared semi-partial correlation for this interaction was 0.05, which means that 5% of the total variability of treatment outcome for physical symptoms is uniquely associated with this interaction.

Table 5 shows the results for predicting reliable and clinical change. Regarding depression, the interaction between the cognitive dimension and chronic medical condition is significant in patients with clinical change and no remission vs. patients with no clinical change and no remission (*OR* = 0.94, 95*%CI* = [0.89, 0.99]). The interaction between the affective dimension and chronic medical condition is significant in patients with clinical change and no remission vs. patients with no clinical change and no remission (*OR* = 1.12, 95*%CI* = [1.00, 1.24]).

To conclude, our results suggest some associations of alexithymia with clinical change and the influence of chronic medical condition on the association between alexithymia and treatment outcome with respect to depression, anxiety, physical symptoms, and general functioning. However, the estimated ORs of ~1.00 suggests that these associations are very small and negligible.

## Discussion

### Main findings

Alexithymia in patients suffering from SSRD was compared to normative data for the general population. The scores of SSRD patients on emotionalizing were lower compared to the norm scores, while elevated scores were found for other BVAQ subscales. The results suggest that SSRD patients show reduced abilities to identify, verbalize, and fantasize, and tend to be aroused by emotional events. This confirms the first hypothesis.

The results also suggest that patients suffering from SSRD improve after multimodal and tailored treatment with regard to anxiety, depression, and physical symptoms (Cohen's *d* ranged 1.21–1.82). This confirms the second hypothesis.

Even though our results suggest some associations of alexithymia with treatment outcome, the odds ratios were close to 1.0 (range 1.02–1.25). Therefore, we render the association of alexithymia not clinically relevant with regard to treatment outcome in terms of depression, anxiety, physical symptoms, and general functioning. This is not what we expected based on the literature.

The cognitive dimension of alexithymia affects treatment outcomes for patients suffering from chronic medical condition but not for patients free of chronic medical conditions with regard to depression and physical symptoms, but odds ratios were equal to 0.95 so we render the association also not clinically relevant. This is not what we expected.

SSRD patients in this study received multimodal treatment tailored to the patients' needs in a SDM model based on repeated PROM, and improved significantly after treatment. However, the level of alexithymia at baseline was not associated with a clinically relevant difference in treatment outcome, although our results suggested that SSRD patients have difficulties with identification and verbalization of emotions. Two possible explanations are the following. First, as treatment outcome was positive independent of suffering from alexithymia, it might be that alexithymia is not a clinically relevant factor needing specific attention when treating patients with SSRD. This might be a possible explanation, if in a randomized clinical trial design the positive association of this multimodal treatment model would be confirmed and again the association with alexithymia would be clinically irrelevant. Second, it could be that the treatment, although yielding positive outcomes, could have better outcomes for patients with high alexithymia scores if the treatment would address them. In that case, treatment should focus on improving identification and verbalization of emotions (6).

Treatment options for SSRD include affective mentalizing as prominent factor because affective mentalization is involved in the onset and prolongation of physical symptomatology and the interpersonal problems that co-occur with these physical symptoms (58, 59). The link between emotional states and bodily distress and how to restore this link could be improved by enhancing ones capacity of emotional awareness. A recent study suggested that women with fibromyalgia might benefit from an emotional disclosure or expression intervention (60). Our results suggested that SSRD patients have difficulties with identification and verbalization of emotions. Therefore, treatment of SSRD patients should focus on improving identification and verbalization of emotions which was also suggested by a previous study (6).

Nevertheless, based on our results we conclude that the influence of alexithymia is clinically irrelevant. Previous studies also found a relationship between alexithymia and interpersonal dysfunction, aggression, and personality disorders (61–63). This association is not yet explored amongst patients suffering from SSRD. Personality characteristics such as interpersonal dysfunction, aggressive behavior or coping strategies may also increase insights in the personal characteristics of patients suffering from SSRD and might offer treatment options. Studies focusing on these kinds of personality characteristics are warranted in order to establish such new therapies. Future studies should also include other patient groups (e.g., depressed patients), to explore differences in emotion regulation between patients having SSRD and other patients. This way, researchers are able to explore whether or not impoverished emotional regulation is a specific feature of SSRD or a common feature of patients suffering from other mental disorders.

### Strengths and limitations of the study

The use of norm scores of a large sample from the general population as a reference group accounts as a major strength, because gender and age-specific norms could be used from a large and representable group. Furthermore, the sample consisted of consecutive patients visiting a Clinical Centre of Excellence for patients with Somatic Symptom Disorder, who were referred by their general practitioner, their medical specialist or their psychiatrist or psychotherapist after an average treatment duration of 7 years without solace. The patients had high complexity levels at biological and psychological symptom levels (64), suffered from comorbidity, complex treatment histories, and high levels of social vulnerability. Hence, this study provides us with findings relevant for such a patient group with SSRD, but results may not necessarily be generalizable to the general population. However, although the composition of patient populations may differ across regions, it is unlikely that the underlying mechanisms of treatment outcome differ across specialty mental health institutions. Unfortunately, our sample was too small to explore the relationship between alexithymia with treatment outcome for different SSRD categories (e.g., somatic symptom disorder and illness anxiety disorder). Our sample was heterogeneous with respect to SSRD diagnoses. Furthermore, we did not include neuropsychological aspects in this study whom may have negatively influence treatment outcome since patients suffering from SSRD experience significant cognitive problems (65).

### Implications for research

Future studies on the relationship between alexithymia and treatment outcome should differentiate between SSRD categories. Future studies should also include large samples and evaluate effectiveness of multimodal tailored treatment supported by SDM and PROM in a randomized design.

## Author contributions

LdV drafted the manuscript. LdV and WE were responsible for design and analysis of the data. WE, KS, and CvdF-C revised the draft. All authors approved of the final manuscript.

### Conflict of interest statement

The authors declare that the research was conducted in the absence of any commercial or financial relationships that could be construed as a potential conflict of interest.

## References

[1] Nemiah JC Sifneos PE. Psychosomatic illness: a problem in communication. Psychother Psychosomat. (1970) 18:154–60. 10.1159/000286074 5520658

[2] Cox BJ Kuch K Parker JDA Schulman ID Evans RJ. Alexithymia in somatoform disorder patients with chronic pain. J Psychosomat Res. (1994) 38:523–7. 10.1016/0022-3999(94)90049-3 7990060

[3] Leweke F Bausch S Leichsenring F Walter B Stingl M. Alexithymia as a predictor of outcome of psychodynamically oriented inpatient treatment. Psychother Res. (2009) 19:323–31. 10.1080/10503300902870554 20183393

[4] Ogrodniczuk JS Piper WE Joyce AS. Effect of alexithymia on the process and outcome of psychotherapy: a programmatic review. Psychiatry Res. (2011) 190:43–8. 10.1016/j.psychres.2010.04.026 20471096

[5] Beck T Breuss M Kumnig M Schüßler G. The first step is the hardest-emotion recognition in patients with somatoform disorders. Zeitschrift für Psychosomatische Medizin und Psychotherapie (2013) 59:385–90. 10.13109/zptm.2013.59.4.385 24307338

[6] Cameron K Ogrodniczuk J Hadjipavlou G. Changes in alexithymia following psychological intervention: a review. Harv Rev Psychiatry (2014) 22:162–78. 10.1097/HRP.0000000000000036 24736520

[7] Gay MC Hanin D Luminet O Effectiveness of an hypnotic imagery intervention on reducing alexithymia. Contemp Hypnos. (2008) 25:1–13. 10.1002/ch.344

[8] Ogrodniczuk JS Sochting I Piper WE Joyce AS. A naturalistic study of alexithymia among psychiatric outpatients treated in an integrated group therapy program. Psychol Psychother. (2012) 85:278–91. 10.1111/j.2044-8341.2011.02032.x 22903919

[9] Tulipani C Morelli F Spedicato MR Maiello E Todarello O Porcelli P. Alexithymia and cancer pain: the effect of psychological intervention. Psychother Psychosomat. (2010) 79:156–63. 10.1159/000286960 20185972

[10] Samur D Tops M Schlinkert C Quirin M Cuijpers P Koole SL. Four decades of research on alexithymia: moving toward clinical applications. Front Psychol. (2013) 4:861. 10.3389/fpsyg.2013.00861 24312069 PMC3832802

[11] Bach M Bach D. Predictive value of alexithymia: a prospective study in somatizing patients. Psychother Psychosomat. (1995) 64:43–8. 10.1159/000288989 7480582

[12] Burba B Oswald R Grigaliunien V Neverauskiene S Jankuviene O Chue P. A controlled study of alexithymia in adolescent patients with persistent somatoform pain disorder. Can J Psychiatry (2006) 51:468–71. 10.1177/070674370605100709 16838829

[13] Cohen K Auld F Brooker H. Is alexithymia related to psychosomatic disorder and somatizing? J Psychosomat Res. (1994) 38:119–27. 8189401 10.1016/0022-3999(94)90085-x

[14] Duddu V Isaac MK Chaturvedi SK. Alexithymia in somatoform and depressive disorders. J Psychosomat Res. (2003) 54:435–8. 10.1016/S0022-3999(02)00440-3 12726899

[15] Kooijman CG The status of alexithymia as a risk factor in medically unexplained physical symptoms. Comprehens Psychiatry (1998) 39:152–9. 10.1016/S0010-440X(98)90075-X 9606582

[16] Kosturek A Gregory RJ Sousou AJ Trief P. Alexithymia and somatic amplification in chronic pain. Psychosomatics (1998) 39:399–404. 10.1016/S0033-3182(98)71298-8 9775696

[17] Moreno-Jiménez B Blanco BL Rodríguez-Muñoz A Hernández EG. The influence of personality factors on health-related quality of life of patients with inflammatory bowel disease. J Psychosomat Res. (2007) 62:39–46. 10.1016/j.jpsychores.2006.07.026 17188119

[18] Taylor GJ Parker JDA Bagby M Acklin MW. Alexithymia and somatic complaints in psychiatric out-patients. J Psychosomat Res. (1992) 36:417–24. 10.1016/0022-3999(92)90002-J 1619582

[19] Verissimo R Mota-Cardoso R Taylor G. Relationships between alexithymia, emotional control, and quality of life in patients with inflammatory bowel disease. Psychother Psychosomat. (1998) 67:75–80. 10.1159/000012263 9556198

[20] Von Rimscha S Moergeli H Weidt S Straumann D Hegemann S Rufer M. Alexithymia and health-related quality of life in patients with dizziness. Psychopathology (2013) 46:377–83. 10.1159/000345357 23296255

[21] Wolf LD Hentz JG Ziemba KS Kirlin KA Noe KH Hoerth MT. Quality of life in psychogenic nonepileptic seizures and epilepsy: the role of somatization and alexithymia. Epilepsy Behav. (2015) 43:81–8. 10.1016/j.yebeh.2014.12.010 25569745

[22] American Psychiatric Association DSM-IV-TR: Diagnostic and Statistical Manual of Mental Disorders, Text Revision. Washington, DC: American Psychiatric Association (2000).

[23] American Psychiatric Association Diagnostic and Statistical Manual of Mental Disorders (DSM-5). Washtingon, DC: American Psychiatric Association (2013).

[24] Black N. Patient reported outcome measures could help transform healthcare. Br Med J. (2013) 346:f167. 10.1136/bmj.f167 23358487

[25] Verhage F Intelligence and Age: Study With Dutch People Aged 12-77. Assen: Van Gorcum (1964).

[26] Van der Feltz-Cornelis CM Andrea H Kessels E Duivenvoorden HJ Biemans H Metz M Shared decision making in combinatie met ROM bij patiënten met gecombineerde lichamelijke en psychische klachten; een klinisch-emperische verkenning [article in Dutch] English: does routine outcome monitoring have a promising future? An investigation into the use of shred decision-making combined with ROM for patients with a combination of physical and psychiatric symptoms. Tijdschrift voor Psychiatrie (2014) 56:375–84. Available online at: http://www.tijdschriftvoorpsychiatrie.nl/issues/478/articles/10323 24953511

[27] Schmand B Lindeboom J Van Harskamp F Dutch Adult Reading Test. Netherlands: Swets & Zeitlinger, Lisse (1992).

[28] Van der Feltz-Cornelis C Swinkels J Blankenstein A Hoedeman R Keuter E. The Dutch multidisciplinary guideline entitled'Medically unexplained physical symptoms and somatoform disorder'. Nederlands tijdschrift voor geneeskunde (2010) 155:A1244–A. 21429250

[29] Van der Feltz-Cornelis CM Hoedeman R Keuter EJ Swinkels JA. Presentation of the Multidisciplinary guideline medically unexplained physical symptoms (MUPS) and Somatoform Disorder in the Netherlands: disease management according to risk profiles. J Psychosomat Res. (2012) 72:168–9. 10.1016/j.jpsychores.2011.11.007 22281461

[30] Vorst HC Bermond B Validity and reliability of the Bermond-Vorst Alexithymia Questionnaire. Pers Individ Diff. (2001) 30:413–34. 10.1016/S0191-8869(00)00033-7

[31] Bermond B Clayton K Liberova A Luminet O Maruszewski T Ricci Bitti PE A cognitive and an affective dimension of alexithymia in six languages and seven populations. Cogn Emot. (2007) 21:1125–36. 10.1080/02699930601056989

[32] Deborde A-S Berthoz S Wallier J Fermanian J Falissard B Jeammet P. The bermond-vorst alexithymia questionnaire cutoff scores: a study in eating-disordered and control subjects. Psychopathology (2007) 41:43–9. 10.1159/000109955 17952021

[33] Müller J Bühner M Ellgring H The assessment of alexithymia: psychometric properties and validity of the Bermond–Vorst alexithymia questionnaire. Pers Individ Diff. (2004) 37:373–91. 10.1016/j.paid.2003.09.010

[34] Zech E Luminet O Rimé B Wagner H Alexithymia and its measurement: confirmatory factor analyses of the 20-item Toronto Alexithymia Scale and the Bermond-Vorst Alexithymia Questionnaire. Eur J Pers. (1999) 13:511–32.

[35] Bagby RM Parker JD Taylor GJ. The twenty-item Toronto Alexithymia Scale–I. Item selection and cross-validation of the factor structure. J Psychosom Res. (1994) 38:23–32. 8126686 10.1016/0022-3999(94)90005-1

[36] Bagby RM Taylor GJ Parker JD. The Twenty-item Toronto Alexithymia Scale–II. Convergent, discriminant, and concurrent validity. J Psychosom Res. (1994) 38:33–40. 8126688 10.1016/0022-3999(94)90006-x

[37] Cronbach LJ Coefficient alpha and the internal structure of tests. Psychometrika (1951) 16:297–334.

[38] Kroenke K Spitzer RL Williams JB. The PHQ-9: validity of a brief depression severity measure. J Gen Inter Med. (2001) 16:606–13. 10.1046/j.1525-1497.2001.016009606.x 11556941 PMC1495268

[39] Kroenke K Spitzer RL Williams JB Löwe B. The patient health questionnaire somatic, anxiety, and depressive symptom scales: a systematic review. Gen Hosp Psychiatry (2010) 32:345–59. 10.1016/j.genhosppsych.2010.03.006 20633738

[40] Spitzer RL Kroenke K Williams JB Löwe B. A brief measure for assessing generalized anxiety disorder: the GAD-7. Arch Int Med. (2006) 166:1092–7. 10.1001/archinte.166.10.1092 16717171

[41] Van Hemert A Lichamelijke Klachten Vragenlijst [in Dutch]. Leiden: Leids Universitair Medisch Centrum (2003).

[42] De Waal MW Van Hemert A Spreadsheet Normscores Dutch Respondents in the General Population and in a General Practioner's Population. Available online at: http://www.psychiatrieweb.mywebhome.nl/pw.somatisatie/files/docs/lkv31norm.pdf (2013).

[43] Ware JE Kosinski M Keller SK SF-36 Physical and Mental Health Summary Scales: A User's Manual. Boston, MA: The Health Institute (1994).

[44] Aaronson NK Muller M Cohen PD Essink-Bot M-L Fekkes M Sanderman R. Translation, validation, and norming of the Dutch language version of the SF-36 Health Survey in community and chronic disease populations. J Clin Epidemiol. (1998) 51:1055–68. 9817123 10.1016/s0895-4356(98)00097-3

[45] Garratt AM Ruta DA Abdalla MI Russell IT. SF 36 health survey questionnaire: II. Responsiveness to changes in health status in four common clinical conditions. Q Health Care (1994) 3:186–92. 10.1136/qshc.3.4.186 10140232 PMC1055239

[46] McHorney CA Ware JE Jr Raczek AE The MOS 36-item short-form health survey (SF-36): II. Psychometric and clinical tests of validity in measuring physical and mental health constructs. Med Care (1993) 13:247–63. 10.1097/00005650-199303000-00006 8450681

[47] Ware J Kosinski M Keller S SF-36 Physical and Mental Health Summary Scales: A Manual for Users of Version 1. 2nd Edn. Lincoln, RI: QualityMetric. Inc (2001).

[48] Maglinte GA Hays RD Kaplan RM. US general population norms for telephone administration of the SF-36v2. J Clin Epidemiol. (2012) 65:497–502. 10.1016/j.jclinepi.2011.09.008 22269331 PMC3582698

[49] Jacobson NS Truax P. Clinical significance: a statistical approach to defining meaningful change in psychotherapy research. J Consul Clin Psychol. (1991) 59:12. 10.1037/0022-006X.59.1.12 2002127

[50] De Vroege L Emons WH Sijtsma K van der Feltz-Cornelis CM. Psychometric properties of the bermond-vorst alexithymia questionnaire (BVAQ) in the general population and a clinical population. Front Psychiatry (2018) 9:111. 10.3389/fpsyt.2018.00111 29740350 PMC5925324

[51] Rosner B Fundamentals of Biostatistics. Nelson Education (2015).

[52] Cohen. J Statistical Power Analysis for the Behavioural Sciences. Hillsdale, NJ: Lawrence Earlbaum Associates (1988).

[53] Cohen J Cohen P West SG Aiken LS Applied Multiple Regression/Correlation Analysis for the Behavioral Sciences. New York, NY: Routledge (2013).

[54] Nagelkerke N. J. D Maximum Likelihood Estimation of Functional Relationships. Berlin: Springer (1992).

[55] IBM Corp IBM SPSS Statistics for Windows, Version 22.0. Armonk, NY: IBM Corp (2011).

[56] Bernaards CA Sijtsma K. Influence of imputation and EM methods on factor analysis when item nonresponse in questionnaire data is nonignorable. Multivar Behav Res. (2000) 35:321–64. 10.1207/S15327906MBR3503_03 26745335

[57] Van Ginkel JR Van der Ark LA Sijtsma K Vermunt JK Two-way imputation: a Bayesian method for estimating missing scores in tests and questionnaires, and an accurate approximation. Comput Stat Data Anal. (2007) 51:4013–27. 10.1016/j.csda.2006.12.022

[58] Luyten P Van Houdenhove B Lemma A Target M Fonagy P A mentalization-based approach to the understanding and treatment of functional somatic disorders. Psychoanalyt Psychother. (2012) 26:121–40. 10.1080/02668734.2012.678061

[59] Tominaga T Choi H Nagoshi Y Wada Y Fukui K. Relationship between alexithymia and coping strategies in patients with somatoform disorder. Neuropsychiatr Dis Treat (2014) 10:55–62. 10.2147/NDT.S55956 24403835 PMC3883553

[60] Geenen R van Ooijen-van der Linden L Lumley MA Bijlsma JW van Middendorp H. The match–mismatch model of emotion processing styles and emotion regulation strategies in fibromyalgia. J Psychosomat Res. (2012) 72:45–50. 10.1016/j.jpsychores.2011.09.004 22200522

[61] Nicolò G Semerari A Lysaker PH Dimaggio G Conti L D'Angerio S. Alexithymia in personality disorders: correlations with symptoms and interpersonal functioning. Psychiatry Res. (2011) 190:37–42. 10.1016/j.psychres.2010.07.046 20800288

[62] Fossati A Acquarini E Feeney JA Borroni S Grazioli F Giarolli LE. Alexithymia and attachment insecurities in impulsive aggression. Attach Hum Dev. (2009) 11:165–82. 10.1080/14616730802625235 19266364

[63] Singleton RA Bruce C Straits, and margaret miller straits. Approach Soc. Res. (1993) 260–77.

[64] Van Eck van der Sluijs JF de Vroege L van Manen AS Rijnders CAT van der Feltz-Cornelis CM. Complexity assessed by the INTERMED in patients with somatic symptom disorder visiting a specialized outpatient mental health care setting: a cross-sectional study. Psychosomatics (2017) 58:427–36. 10.1016/j.psym.2017.02.008 28347505

[65] de Vroege L Timmermans A Kop WJ van der Feltz-Cornelis CM Neurocognitive dysfunctioning and the impact of comorbid depression and anxiety in patients with somatic symptom and related disorders: a cross-sectional clinical study. Psychol Med. (2017) 4:1–11. 10.1017/S0033291717003300 29198246

